# Life-time HIV testing among people who inject drugs in Iran: results from the National Rapid Assessment and Response survey

**DOI:** 10.3389/fpubh.2023.1253407

**Published:** 2023-10-17

**Authors:** Payam Roshanfekr, Salah Eddin Karimi, Sakineh Narouee, Leila Moftakhar, Meroe Vameghi, Delaram Ali, Peter Higgs, Neda Soleimanvandiazar

**Affiliations:** ^1^Social Welfare Management Research Center, Social Health Research Institute, University of Social Welfare and Rehabilitation Sciences, Tehran, Iran; ^2^Social Determinants of Health Research Center, Health Management and Safety Promotion Research Institute, Tabriz University of Medical Sciences, Tabriz, Iran; ^3^Department of Statistics and Epidemiology, Faculty of Health, Kerman University Medical of Sciences, Kerman, Iran; ^4^Research Committee, Shiraz University of Medical Sciences, Shiraz, Iran; ^5^Department of Public Health, La Trobe University, Melbourne, VIC, Australia; ^6^Behaviours and Health Risks Program, Burnet Institute, Melbourne, VIC, Australia; ^7^Preventive Medicine and Public Health Research Center, Psychosocial Health Research Institute, Iran University of Medical Sciences, Tehran, Iran

**Keywords:** HIV testing, people who inject drugs, PWID, rapid assessment and response, Iran

## Abstract

**Background:**

HIV testing is recommended for people who inject drugs (PWID). The aim of this study was to determine the prevalence of lifetime HIV testing among PWID and to better understand the predictors for HIV testing in a convenience sample across Iran.

**Materials and methods:**

This study is a secondary analysis of Iran’s National Rapid Assessment and Response survey conducted between October 2016 and March 2017. Analysis includes the 999 people who reported injecting drugs across the capital cities of 21 provinces. Data were collected by using the venue-based application of the Time Location Sampling (TLS) frame. Chi-square tests were used to examine the difference between HIV testing across different quantitative variables. Logistic regression was used to determine the predictors of life-time HIV testing. Analysis was performed using STATA V.12 software with a significance level of 95%.

**Results:**

Most participants were male (*n* = 902, 93.50%), and over half (*n* = 555, 59.17%) were older than 35 years old. About one-third, (*n* = 326, 38%) of people interviewed were single and another one-third (*n* = 251, 29%) reported being divorced. Over two-thirds of participants (*n* = 678, 69.78%) in this study reported lifetime HIV testing. The results from a multiple variable logistic regression showed people with a university education were more likely to have been previously tested for HIV than illiterate people (OR = 18.87, 95%CI 2.85–124.6, value of *p* = 0.002). Those individuals who reported ever receiving methadone treatment were 2.8 times more likely to have been tested for HIV than individuals without methadone treatment experience (OR = 2.89, 95%CI 1.53–5.42, value of *p* < 0.001). Needle syringe sharing in last month, was negatively associated with life-time HIV testing (OR = 0.29, 95%CI 0.17–0.48, value of *p* < 0.001).

**Conclusion:**

Despite Iran’s wide availability and access to counseling services for HIV testing in key populations, the proportion of PWID being tested for HIV could be improved. Developing effective strategies to increase people’s understanding and awareness of the importance of and need for HIV prevention and familiarity with HIV testing sites is an essential step in increasing HIV testing for this population. Studies on more recent HIV testing are required to better assess and understand the frequency of HIV testing among PWID in Iran.

## Introduction

In many countries, the human immunodeficiency virus (HIV) epidemic is concentrated within subgroups of people that the Joint United Nations Program on HIV/AIDS (UNAIDS) call “key populations.” These populations include female sex workers (FSWs), transgender individuals, people who inject drugs (PWID), men who have sex with men (MSM), and people with prison histories (1–3). While these key populations comprise a small proportion of the general population they are known to be at higher risk of acquiring HIV infection (4).

According to the World Health Organization (WHO) (4) in 2020, individuals from key population groups and their sexual partners accounted for almost two-thirds of all new HIV infections globally and higher proportions across eastern European, central Asian, Middle Eastern, and North African regions (5). Available data suggest that the risk of acquiring HIV among PWID was 35 times higher than those who do not inject drugs (6–8).

UNAIDS has set ambitious 90–90-90 targets, where 90% of all people living with HIV should know their HIV status, 90% of people with diagnosed HIV should receive antiretroviral therapy, and 90% of all people receiving antiretroviral therapy should have viral suppression. This paves the way for viral suppression and is an important foundation for meeting the HIV/AIDS elimination goal by 2030 (9, 10).

HIV prevention tools including sterile needle and syringe programs, medically assisted therapy, HIV counseling and testing, and antiretroviral therapy have the strongest effects on reducing the risk of acquiring HIV among PWID. HIV testing is a cost-effective strategy because when people are diagnosed, they are linked to treatment and can achieve viral suppression, and reduce the risk of transmission of HIV even when people are engaged in risky behaviors (11). In addition, when people find out that they have tested HIV positive, research suggests that they are more likely to use sterile injecting equipment and condoms when having sex, thus reducing the risk of transmitting HIV to others (12–14).

However, despite access to free HIV testing services, and frequent recommendations for PWID to test at least yearly, the proportion of current and life-time HIV testing in Iran is still less than optimal (with 2010 data reporting 24.9 and 49.8% respectively) (15). As well, the proportion of current HIV testing among PWID in the United States was reported to be 47 and 53%, respectively (16, 17). Fear of HIV testing, stigma in the community, low perceptions of HIV risk, and other socio-cultural factors have been found to lead to lower proportions of PWID testing for HIV (15, 18, 19).

Iran is one of the most populated countries in the Middle East with a concentrated HIV epidemic among PWID (15). According to the Ministry of Health, data reported to UNAIDS, there were 90,000 PWID in 2021 in Iran (20). PWID prevalence in the Iranian general population is high with estimates of 0.28% and recent studies show HIV prevalence of 9.7% among PWID (95% CI = 7.6–11.9) (21). Despite HIV testing sites being free and widely available for key populations in Iran, previous research indicates that HIV testing in PWID remains inadequate (15). The most recent study measuring HIV testing among the PWID was conducted on male PWID by Shokoohi et al. across 10 provinces of Iran in 2010 (15). Since then, very little has been documented about HIV testing in Iranian PWID and its correlated factors. Monitoring the health status of this population will help prevent transmission of HIV to other people. This study aimed to determine life-time HIV testing for PWID and to identify factors associated with testing among PWID in Iran.

## Methodology

### Study design

Secondary data analysis of survey data collected by the Social Welfare Management Research Center for the National Rapid Assessment and Response (RAR) study on HIV/AIDS-related Risky Behaviors among street based female sex workers and street based PWID in Iran was conducted.

### Setting and participation

The RAR study was conducted over 20 weeks between October 2016 and March 2017 across the capital cities of 21 provinces in Iran. A total of 2,310 people were recruited randomly using venue-based application of Time Location Sampling (TLS) (22–25).

In this sub-study, those included were: aged 18 or over, currently (in last 3 months) residing in a provincial capital city, reporting ever drug injecting and providing consent to participate. When including only eligible participants, 999 PWID were included. Participants were excluded if they did not provide responses to the questions for the main outcome of the study (i.e., HIV testing) ultimately 969 people remained in the final analysis.

Participants were recruited using the venue-day-time (VDT) sampling method, which is based on place and time (26). Place refers to hangouts and neighborhoods where the target population can be found and time refers to specific days and periods of time when the target population gathers in each space. These spaces and days are classified into standard space–time sections (four-hour intervals per space) and are known as venue-day-time or VDT units.

### Variables, data sources/measurement

#### Dependent variable: reporting life-time HIV testing

The main outcome of interest for this study was to find factors related to life-time HIV testing in PWID, accordingly participants were asked “have you ever been tested for HIV” and responses to the question were coded as Yes or No.

#### Independent variables

The variables selected as influencing predictors were based on previously published literature (22) and the primary study objectives of lifetime HIV testing and included: sex, age at interview, educational level, marital status, employment status, having a history of arrest or imprisonment, having reported injecting drugs while in prison, previous methadone treatment, reporting needle syringe sharing in last month, ever had commercial sex, having used condoms during the last sex, ever consumed alcohol, simultaneous consumption of alcohol while having sex, having genital warts in the previous year, having genital secretions in the previous year.

The questions of this self-report survey were based on previous questionnaires developed by the Regional Knowledge Hub, and the WHO Collaborating Centre for HIV Surveillance, Institute for Futures Studies in Health, Kerman University of Medical Sciences (27).

### Ethical approval

The study protocol and procedures were reviewed and approved by the Human Research Ethics Committee and the Research Review Board at the University of Social Welfare and Rehabilitation Sciences (IR.USWR.REC.1394.392).

All participants were provided with information on the aims and objectives of the study, and written informed consent was obtained from all participants prior to their survey completion.

### Statistical analysis

Lifetime HIV testing was calculated in total for different subgroups (sex, age, marital status, education level) of the PWID recruited. The frequencies were reported for categorical variables. The Chi-square test and Fisher’s exact test were used to examine the differences between the status of lifetime HIV testing among different variables. Bivariate and multivariate logistic regression were used to determine the factors affecting lifetime HIV testing. Finally, predictor variables selected from independent variables, were kept in our final regression models according to the results of statistical testing (Table 1). In addition, multicollinearity between the four variables was evaluated using VIF. Variables with *P* (value) equal and less than 0.2 in the Bivariate regression logistic were entered into the multivariate logistic regression model. Odds ratios (OR) along with 95% confidence intervals (95%CI) are reported. All analyses were performed using STATA V.12.

**Table 1:** Distribution of variables based on the status of lifetime HIV testing among PWID in 2017 in Iran.

Value	Lifetime HIV testing				Value of p*
	No	%	Yes	%	
Sex					
Female	23	36.5	40	63.5	0.24
Male	268	29.6	638	70.4	
Age					
18–24	23	41.1	33	58.9	<0.001
25–34	124	37.9	203	62.1	
35≥	125	22.5	430	77.5	
Marital status					
Single	118	36.2	208	63.8	0.02
Married	29	26.1	82	73.9	
Separate	30	30.0	70	70.0	
Sigeh (temporary marriage)	11	32.4	23	67.6	
Divorced	58	23.1	193	76.9	
Widow	9	24.3	28	75.7	
Education level					
Never attended school	34	40.0	51	60.0	0.13
Basic or Primary school (1–6 years of education)	75	29.4	180	70.6	
Guidance school (7–9 years of education)	93	26.6	257	73.4	
Diploma or High school (10–12 years of education)	72	32.0	153	68.0	
University education	12	25.0	36	75.0	
Employment status					
Full time/permanent job	11	23.9	35	76.1	0.001
Part time/temporary job	80	27.5	211	72.5	
Family support	34	43.0	45	57.0	
Other sources (GO/NGO support)	63	24.7	192	75.3	
No paid work	90	37.7	149	62.3	
Ever arrested or incarcerated					
No	73	38.4	117	61.6	0.002
Yes	205	27.1	552	72.9	
Ever injected in prison					
No	126	22.5	434	77.5	<0.001
Yes	78	41.5	110	58.5	
Needle syringe sharing in last month					
No	122	26.2	343	73.8	<0.001
Yes	88	47.1	99	52.9	
Ever receiving methadone treatment					
No	204	31.9	435	68.1	0.07
Yes	81	26.2	228	73.8	
Commercial sex ever					
No	116	27.6	304	72.4	0.2
Yes	129	31.6	279	68.4	
Condom used at last sex					
No	142	31.6	307	68.4	0.01
Yes	80	23.8	256	76.2	
Ever consumed alcohol					
No	76	32.8	156	67.2	0.34
Yes	206	29.5	493	70.5	
Ever consumed alcohol & having sex concurrently					
No	19	20.7	73	79.3	0.58
Yes	20	24.1	63	75.9	
Genital warts (previous year)					
No	211	26.9	573	73.1	0.01
Yes	49	37.7	81	62.3	
Genital secretions (previous year)					
No	184	26.1	522	73.9	0.01
Yes	58	35.8	104	64.2	

^*^Value of *p* for chi-square test.

## Results

### Characteristics of participants

Most participants were male (*n* = 902, 93.50%), and over half (*n* = 555, 59.17%) were older than 35 years old. About one-third, (*n* = 326, 38%) of people interviewed were single and another one-third (*n* = 251, 29%) reported being divorced. One-third (*n* = 450, 36%) had guidance school education (age 14 years) and (*n* = 291, 39%) of participants reported part-time/ temporary employment.

The highest proportion of life-time HIV testing was found in participants who reported ever being arrested or having incarceration histories (*n* = 552, 82.51%), and those who reported ever consuming alcohol (*n* = 493, 75.96%). Almost two-thirds (*n* = 430, 64.56%) of participants who were older than 35 years, reported life-time HIV testing.

### Prevalence of lifetime HIV testing

Over two-thirds of participants (*n* = 678, 69.78%) in this study reported previous HIV testing.

### Association between lifetime HIV testing and socio-demographic characteristics

Life-time HIV testing in participants was correlated with age, marital status, employment status, having a history of imprisonment, reporting injecting drugs while in prison, and reporting the use of condoms at their most recent sexual experience (*p* < 0.05) (Table 1).

In the multivariate logistic regression model, results indicated significant differences between those who reported lifetime HIV testing and those who did not, in terms of education level. Life-time HIV testing was significantly higher in PWIDs with a university education than in those with no schooling ((noting the wide confidence interval) OR = 18.87, 95% CI 2.85–124.6, value of *p* = 0.002). The PWID in the study who reported ever receiving methadone treatment were 2.8 times more likely to have previous HIV testing than individuals who reported never experiencing methadone treatment (OR = 2.89 95% CI 1.53–5.42, value of *p*<0.001). Finally, those participants who reported having shared a needle/syringe in the last month were much less likely to also report having ever been tested for HIV compared to those not having shared (OR = 0.29, 95% CI 0.17–0.48, value of *p* < 0.001) (Table 2).

**Table 2:** Predictive variables of Lifetime HIV Testing among PWID based on logistic regression results in 2017 in Iran.

Variables		Crude OR (CI 95%)	Value of *p*	Adjust OR (CI 95%)	Value of *p*
Education level	Never attended school	-	-	-	-
	Basic or Primary school (1–6 years of education)	1.85 (1.04, 3.27)	0.03	1.72 (0.63, 4.67)	0.28
	Guidance school (7–9 years of education)	2.18 (1.25, 3.79)	0.006	1.81 (0.67, 4.88)	0.23
	Diploma or High school (10–12 years of education)	1.80 (1.00, 3.22)	0.04	0.88 (0.31, 2.52)	0.82
	University education	2.94 (1.12, 7.74)	0.02	18.87 (2.85, 124.6)	0.002
Needle syringe sharing in last month	No	-	-	-	-
	Yes	0.39 (0.26, 0.58)	0.001	0.29 (0.17, 0.48)	0.001
Ever receiving methadone treatment	No	-	-	-	-
	Yes	1.31 (0.92, 1.86)	0.12	2.89 (1.53, 5.42)	0.001

## Discussion

Based on the results of our study, more than two-thirds of the participants (*n* = 678, 69.78%) had been ever tested for HIV and most of those who had were over 35 years old, single, and a history of imprisonment.

Previous research showed that the prevalence of lifetime HIV testing in Iranian PWID ranged from 27.4% in 2007 (28), to 49.8% in 2010 (15). Other studies in international settings show rates of previous HIV testing to be between 33 and 87.7% (12, 15, 17, 29–31). Previous international literature shows that the diverse social and cultural settings, HIV-related stigma and discrimination and civil unrest, are all likely to have a negative impact on access to HIV testing services and therefore on subsequent HIV testing (32–37).

To overcome barriers and maintain access to HIV testing during times of crisis, such as global pandemics like COVID-19, healthcare systems and clinicians, have to consider expanding HIV programs, including HIV self-testing kits, telehealth, mobile HIV testing sites, and routine opt-out screening within health care settings (38).

In this study, HIV testing in people who reported having ever received methadone treatment was 2.8 times more likely than in people without a history in methadone treatment. The results of our study were consistent with the results of research in Myanmar showing a direct relationship between HIV testing and drug treatment (12). This is further supported by data from a systematic review and meta-analysis with data from ten studies, showing positive associations between having ever been on opioid agonist therapy and previous HIV testing (29). HIV testing in such circumstances may, however, not be voluntary and reflects the higher levels of surveillance that some populations of PWID are impacted by. Also, people in drug treatment may be in the position to refocus their health priorities away from drug withdrawal and may therefore make a more conscious decision to test for HIV.

The results of our study show a correlation between educational level and life-time HIV testing which is supported by previous studies in China and Tehran, where increasing education levels also correlated with increased rates of HIV testing. However, the wide confidence intervals mean our results should be interpreted with caution (39, 40). It may be that education provides people with increased understanding about HIV risk and the importance of HIV infection prevention. Education and increased health literacy may increase an individuals’ willingness to get tested but we are aware that this is likely also influenced or modified by other variables such as social class, income, stable housing and so on. Caution should be taken when using this finding on its own to develop HIV prevention interventions for PWID. HIV testing rates are more likely to be improved through systemic interventions (41), such as increasing people’s awareness and increasing access to voluntary routine opt-out testing within primary health care services and prison settings (42, 43).

### Implication and recommendations

According to the World Health Organization, the most important interventions for key populations include primary health care where HIV testing is routine (44). The explosive epidemics of HIV in Iran, resulted in the development of harm-reduction-focused intervention programs including methadone and needle syringe programs specifically targeting key populations as early as 2002. However, despite the wide availability of these harm reduction centers in Tehran, relatively low proportions of PWID people report having used these services (28) and the coverage of HIV testing in PWID remains inadequate (28). Previous studies have shown that illegal nature of drug injecting, as well as social and geographic issues, social stigma, difficulty in accessing services, the limited unavailability of these centers, or the restricted opening and closing hours of these centers, altogether lead to less than optimum numbers of people using harm reduction services (45, 46). One intervention adopted in recent years is to initiate a provider-initiated HIV testing strategy rather than relying on PWID themselves to present and ask for testing (15). Our data suggest that mobile HIV testing sites and harm reduction services should be made more accessible in areas where PWIDs commonly gather.

Improving the quality of counseling in health service centers targeting PWID is another strategy that is known to increase HIV testing. Regular testing has been found to increase the early diagnosis of HIV in these individuals and provides support that can guide them to receive appropriate medical care including antiretroviral treatment (47).

The role of harm reduction centers that work daily with PWID is an important part of providing accurate and up-to-date information about HIV testing locations and increasing risk awareness (48). Therefore, to overcome these obstacles, public health policymakers in Iran need to also develop interventions that help to eliminate stigma and fear among health care providers who regularly work with PWID.

### Strengths and limitations

One of the strengths of the present study is the large sample size of PWID at the national level, where participants were selected with a sophisticated sampling framework. However, this study has some limitations. Firstly, the study is cross-sectional, therefore, we cannot infer any causal relationship between the factors affecting life-time HIV testing in study participants. In this study, only lifetime HIV testing was reported. The reliance on participants’ self-report about HIV testing means that their responses may be affected by recall bias, social desirability bias and accordingly the results may be underestimated or overestimated. Participants in this study were all recruited from the street on a venue-based application of Time Location Sampling, across 21 capital cities of Iranian provinces, so one should be careful in generalizing the findings. Samples of PWID who are more home based or who frequent drop-in centers in other Iranian counties may well be very different. As well, in this study, few younger participants were recruited, which could reflect differences in street-based consumption patterns. For these reasons, the results should be interpreted with caution.

## Conclusion

Despite the existence of care, counseling, and treatment services for key populations, the coverage of HIV testing in PWID is inadequate. Given the barriers to HIV testing in this group, public health policymakers need to develop specific interventions that can help people injecting in street-based settings to overcome these barriers, including increasing people’s awareness of the importance of HIV prevention, promoting awareness of HIV testing sites and other available services.

## Data availability statement

The raw data supporting the conclusions of this article will be made available by the authors, without undue reservation.

## Ethics statement

The study protocol and procedures were reviewed and approved by the Human Research Ethics Committee and the Research Review Board at the University of Social Welfare and Rehabilitation Sciences (IR.USWR.REC.1394.392). All participants were provided with information on the aims and objectives of the study, and written informed consent was obtained from all participants prior to their survey completion. The studies were conducted in accordance with the local legislation and institutional requirements. Written informed consent for participation in this study was provided by the participants’ legal guardians/next of kin.

## Author contributions

PR: Conceptualization, Formal analysis, Methodology, Project administration, Supervision, Writing – original draft, Writing – review & editing, Funding acquisition, Investigation, Resources. SK: Formal analysis, Methodology, Writing – original draft, Writing – review & editing, Data curation, Software, Validation. SN: Data curation, Formal analysis, Methodology, Software, Writing – original draft, Writing – review & editing. LM: Data curation, Formal analysis, Methodology, Software, Writing – original draft, Writing – review & editing. MV: Writing – review & editing, Conceptualization, Investigation, Project administration, Supervision, Validation, Visualization. DA: Conceptualization, Investigation, Project administration, Visualization, Writing – review & editing, Data curation. PH: Visualization, Writing – review & editing, Methodology, Supervision, Validation. NS: Methodology, Supervision, Writing – review & editing, Conceptualization, Formal analysis, Project administration, Writing – original draft.

## Abbreviations

RAR: Rapid assessment & response
PWIDs: People who inject drugs
HIV: Human Immunodeficiency Viruses
AIDS: Acquired Immunodeficiency Syndrome
UNAIDS: Joint United Nations Program on HIV/AIDS
FSWs: Female sex workers
MSMs: Men who have sex with men
WHO: World Health Organization
NSP: Needle and syringe programs
MAT: Medically assisted therapy
HCT: HIV counseling and testing
ART: Antiretroviral therapy
VDT: Venue-day-time
TLS: Time location sampling
CI: Confidence intervals
AOR: Adjusted odds ratios
COR: Crude odds ratios
